# Strong Association between Serological Status and Probability of Progression to Clinical Visceral Leishmaniasis in Prospective Cohort Studies in India and Nepal

**DOI:** 10.1371/journal.pntd.0002657

**Published:** 2014-01-23

**Authors:** Epco Hasker, Paritosh Malaviya, Kamlesh Gidwani, Albert Picado, Bart Ostyn, Sangeeta Kansal, Rudra Pratap Singh, Om Prakash Singh, Ankita Chourasia, Abhishek Kumar Singh, Ravi Shankar, Mary E. Wilson, Basudha Khanal, Suman Rijal, Marleen Boelaert, Shyam Sundar

**Affiliations:** 1 Institute of Tropical Medicine, Antwerp, Belgium; 2 Institute of Medical Sciences, Banaras Hindu University, Varanasi, India; 3 Department of Biotechnology, University of Turku, Turku, Finland; 4 Barcelona Centre for International Health Research (CRESIB, Hospital Clínic-Universitat de Barcelona), Barcelona, Spain; 5 University of Iowa and the Veterans' Affairs Medical Center, Iowa City, Iowa, United States of America; 6 B.P. Koirala Institute of Health Sciences, Dharan, Nepal; The Ohio State University, United States of America

## Abstract

**Introduction:**

Asymptomatic persons infected with the parasites causing visceral leishmaniasis (VL) usually outnumber clinically apparent cases by a ratio of 4–10 to 1. We assessed the risk of progression from infection to disease as a function of DAT and rK39 serological titers.

**Methods:**

We used available data on four cohorts from villages in India and Nepal that are highly endemic for *Leishmania donovani*. In each cohort two serosurveys had been conducted. Based on results of initial surveys, subjects were classified as seronegative, moderately seropositive or strongly seropositive using both DAT and rK39. Based on the combination of first and second survey results we identified seroconvertors for both markers. Seroconvertors were subdivided in high and low titer convertors. Subjects were followed up for at least one year following the second survey. Incident VL cases were recorded and verified.

**Results:**

We assessed a total of 32,529 enrolled subjects, for a total follow-up time of 72,169 person years. Altogether 235 incident VL cases were documented. The probability of progression to disease was strongly associated with initial serostatus and with seroconversion; this was particularly the case for those with high titers and most prominently among seroconvertors. For high titer DAT convertors the hazard ratio reached as high as 97.4 when compared to non-convertors. The strengths of the associations varied between cohorts and between markers but similar trends were observed between the four cohorts and the two markers.

**Discussion:**

There is a strongly increased risk of progressing to disease among DAT and/or rK39 seropositives with high titers. The options for prophylactic treatment for this group merit further investigation, as it could be of clinical benefit if it prevents progression to disease. Prophylactic treatment might also have a public health benefit if it can be corroborated that these asymptomatically infected individuals are infectious for sand flies.

## Author Summary

To study the association between positive serology for VL among asymptomatic persons and progression to disease, we analyzed combined data from two different cohort studies in which over 32,000 subjects were enrolled. All subjects were screened for presence of antibodies to *L. donovani* with DAT and rK39. After an interval of 6–12 months, a second round of surveys was conducted in which seroconvertors were identified, i.e. persons seronegative at baseline that had become seropositive in the interval between surveys. We subdivided the subjects according to serologic titers scored as negative, moderately positive or strongly positive. During follow-up we observed that the risk of progressing to disease was not very much different between seronegative and moderately seropositive subjects, but strongly increased for high titer seropositive subjects. This pattern was true for the initial serology, and even more pronounced among seroconvertors. Given the strongly increased risk of progression to disease among those with high DAT or rK39 titers, these data raise the question whether prophylactic treatment would be beneficial. The case for prophylactic treatment would be further strengthened if it could be shown that asymptomatically infected individuals are infectious to sand flies.

## Introduction

Visceral leishmaniasis (VL) is a vector borne infectious disease that is endemic in parts of Asia, Africa, South America and Europe, and is caused by different species of the genus *Leishmania*. In South America and the Mediterranean region, the disease is mainly caused by *Leishmania infantum*, which is a parasite of dogs opportunistically transmitted to humans. In East Africa and on the Indian subcontinent the disease is caused by *L. donovani*. *L. donovani* is essentially a parasite of humans, though recently there have been some reports of a possible animal reservoir [1]. Insect vectors are different species of phlebotomine sand flies. The symptomatic disease VL is a severe systemic disease characterized by fever, hepatosplenomegaly, pancytopenia and cachexia. VL has a protracted course and ultimately leads to death if it is not treated [2]. However, as in tuberculosis, not every infected individual develops disease, and there is a spectrum ranging from asymptomatic infection and subclinical disease to the full-blown VL syndrome [3]. Subclinical or mild forms of visceral disease have been described due to *L. infantum* in a Brazilian study, and due to *L. tropica* in veterans of Operation Desert Storm [4], [5]. Epidemiological surveys showed that asymptomatic and subclinical forms are frequent and outnumber clinical VL cases [6], . The prevalence of asymptomatic infections of *L. infantum* varied from 0.6 to 71% in endemic areas [6]. Prospective studies cite ratios of incident asymptomatic infections with *L. donovani or L. infantum* to symptomatic clinical cases as 1∶2.4 in Sudan, 4∶1 in Kenya, 5.6∶1 in Ethiopia, 6∶1 to 18∶1 in Brazil, 4∶1 in Bangladesh and 8.9∶1 in India and Nepal [8], [9], [10], [11], [12]. These large numbers of latent leishmania infections raise the questions on whether there is any clinical relevance of asymptomatic infection for an individual, and whether these latent infections play any role in disease transmission. If asymptomatically infected persons are indeed infectious for sand flies, theoretically they could contribute to an epidemic [13].

Studies of the risk of progression from infection to disease have yielded contradictory results. In a study by Das *et al.* 21 out of 91 polymerase chain reaction (PCR) and/or recombinant K39 (rK39) positive but asymptomatic individuals (23.1%) progressed to clinical VL over a follow-up period of one year [14]. Singh *et al.* reported 38 VL cases among 55 asymptomatic seropositive subjects followed up for one year (69%) [15]. Gidwani *et al.* followed up a cohort of 870 endemic subjects of whom 230 were Direct Agglutination Test (DAT) positive at onset and 120 were rK39 positive. Over a 2-year follow-up, 15 VL cases occurred among 583 persons negative on both tests (2.6%) versus 10 cases among 287 DAT and/or rK39 positive individuals (3.5%); the difference was not statistically significant [16]. Ostyn *et al.* followed up a cohort of 9034 DAT negative subjects in high VL incidence villages in India and Nepal. The ratio of incident infection to incident disease was 8.9 to 1, the risk of disease was 11.5 times higher (95% confidence interval 4.5–28.3) in those that had converted from seronegative to seropositive during the first year when compared to the risk of those that remained seronegative at the start of follow-up [7]. The absolute risk was not very high though, with 7 out of 375 seroconvertors (1.9%) progressing to disease. Two studies from Brazil found no association between positive results in serological tests among asymptomatic persons and progression to VL disease [17] [18].

Whereas the antibody-positive cases reported by Gidwani *et al.* included a mixture of individuals with remote and recent seroconversion, Ostyn *et al.* were able to establish an association between positive serology and disease in individuals who were recently infected (recent seroconversion). Similarly, the asymptomatic subjects reported upon by Singh *et al.* were contacts of patients with VL, and as such they may have been recently infected and at higher risk of progression to disease. There has been recent controversy about the role of various antibody tests as markers of infection, and about the link between serologic status and disease risk in humans [19] [20]. To explore the association between serologic status and disease risk, we analyzed prospective cohort data from two population-based research projects in endemic areas of India and Nepal. The main objective was to estimate the risk of progression to disease in asymptomatically infected persons and particularly among recent seroconvertors.

## Methods

### Study populations

We pooled prospectively obtained data from two population-based research projects focused on the epidemiology of VL in India and Nepal. The data were divided into four distinct cohorts. The first two cohorts belong to the KALANET research project which was concluded in India and Nepal in 2009; the two other cohorts belong to the NIH/TMRC research project that started in 2008 in India [21] [22].

The main aim of the KALANET project was to investigate the effectiveness of insecticide-treated bed nets (ITN) as an intervention against VL. A paired-cluster randomized trial was conducted in 26 highly VL endemic villages in the Muzaffarpur district of Bihar, India (n = 16 clusters) and the Terai region of Nepal (n = 10 clusters). In the 13 intervention villages, ITN were distributed at the start of the trial, and the incidence rate of leishmania infection and VL in those villages was compared with that in 13 non-intervention villages over a two-year period. Incident infection, measured by seroconversion according to the DAT, was the primary outcome measure. Importantly, no differences in primary outcome were found between intervention and non-intervention villages, (seroconversion 5.4% vs. 5.5%), nor in the secondary outcome, incident VL (OR 0.99) [21]. During the current study we used available data from the 13,286 subjects who had been enrolled in the baseline survey: 7,950 from India (cohort 1) and 5,336 from Nepal (cohort 2).

In the framework of the NIH/TMRC research project, a population of approximately 85,000 persons living in 50 rural villages of Muzaffarpur district, a highly VL endemic district in Bihar India, have been under active surveillance since April 2008. From this population, hereafter called ‘TMRC - old area’, data are available for 13,134 subjects enrolled in the baseline serosurvey conducted in the villages with the highest VL incidence in the 3-year period preceding the survey (cohort 3). In 2010 the study population was expanded to an additional 10 villages named ‘TMRC-new area’. The additional villages were selected based on high reported VL incidence during the year preceding their entry. All subjects providing informed consent were included in a serosurvey. Data for 7,934 individuals from this ‘TMRC-new area’ (Cohort 4) were included in the present study.

Altogether we thus have serology data available on 34,354 individuals enrolled in one of the four cohorts with a total of 76,113 person years follow-up. HIV prevalence in the area is assumed to be very low. In the TMRC study populations HIV testing was conducted among a subgroup of 1,787 persons of whom only 4 (0.2%) tested positive.

### Study procedures

Study procedures are extensively described in Picado et al (2010) and Hasker et al (2012) [21] [22]. Briefly, the KALANET project conducted three sero surveys separated by 12 month intervals starting in November 2006. Two sero surveys were conducted in each of the TMRC areas, separated by a 12 month interval in the ‘old area’, and a 6-month interval in the ‘new area’. The TMRC baseline surveys took place between December 2009 and February 2010 in the ‘old area’, or in April–May 2011 in the ‘new area’. During all surveys, each subject aged over 2 years and residing in the selected villages was asked for a capillary blood sample collected by finger prick and stored on filter paper. These samples were tested with DAT and rK39 ELISA. Since enrolment, all subjects have been under regular follow-up by a network of village volunteers and have been visited on an annual basis as part of household surveys. Follow-up ended in May 2009 for the KALANET cohorts and is still ongoing for the TMRC cohorts.

DAT and rK39 ELISA assays of serologic status were done from eluted filter paper blood as described elsewhere in the same laboratory at BHU India for the three Indian cohorts (Cohorts 1, 3 and 4) and at BPKIHS Nepal for the Nepal samples (Cohort 2) [23]. For the purpose of this analysis, DAT seropositivity was defined as a DAT end titer of 1∶1,600 or above. Seropositives were again subdivided into moderately seropositive (DAT titer ≥1∶1,600 & <1∶25,600) and highly sero-positive (DAT titer ≥1∶25,600). A seroconvertor was defined as an individual who was DAT negative in the baseline survey but DAT positive in the follow-up survey with an increase of at least 2 titer steps. DAT seroconvertors were also subdivided into moderate and high using the cut offs described above. For subjects from the KALANET study (cohorts 1 and 2) we considered only the follow-up results of the second round of serosurvey and not those of the third round to define DAT seroconversion.

For the KALANET cohort (Cohorts 1 and 2) results for rK39 were available from the baseline survey only. The cut-off for a seropositive test for rK39 ELISA was determined for the 4 cohorts separately, using the percentage point optical density (OD, set at 100% for a positive control) with the highest Youden index as a cut off with recent VL cases (diagnosed in the 24 months period preceding the survey) as reference. As a second cut off point, to distinguish moderately seropositive from highly seropositive, we used the mean OD plus two standard deviations among those who had never suffered VL at the time of the baseline surveys. A seroconvertor was defined as an individual who was rK39 negative in the baseline survey but rK39 positive in the follow-up survey with an increase in optical density of at least three percentage points. RK39 seroconvertors were also subdivided into moderate and high, using the cut offs described above for seropositivity.

A case of VL was defined as a person with a clinical history typical for VL (fever of more than 2 weeks' duration, not responding to anti malaria treatment) and in whom the presence of *L. donovani* has been confirmed; or a person with the combination of a clinical history typical for VL and a positive result of the rK39 test (Inbios International, Seattle, WA, USA) and a good response to specific VL treatment. All cases reported were ascertained by study physicians.

### Data analysis

Data were analyzed in Stata/IC V10.1 (Stata Corp., College Station Tx, USA). For analytical purposes we considered the four cohorts pooled and separately. Kaplan-Meier curves of DAT or rK39 seropositive or seroconversion titer levels were made for the combined study population. Cox regression models were fitted to each cohort separately and the combined cohorts to test for (1) associations between initial DAT or rK39 sero-positivity and clinical VL, and for (2) associations between DAT or rK39 seroconversion and clinical VL. The latter was done only for the TMRC cohorts (cohort 3 and 4) since for the KALANET cohorts (cohort 1 and 2) rK39 results were only available at baseline. In the models for combined cohorts, the factor ‘cohort’ was included as a fixed effect. We tested for interactions, and if there were no interactions we presented a combined effect for the different study cohorts. During follow-up visits, information on all household members was obtained irrespective of whether they were present or absent. Deaths were censored on the reported date of decease.

### Ethical considerations

Both research projects (KALANET and TMRC) sought consent at three levels: community, household and individual. Communities were duly informed about the purpose of the studies and consent was sought from village leaders. For the KALANET trial ethical clearance was obtained from the ethical committees of BHU (India), the BPKIHS (Nepal), the London School of Hygiene and Tropical Medicine (UK), and the University of Antwerp (Belgium). For the TMRC study ethical clearance was obtained from the review committee of the U.S. National Institutes of Health (NIH), as well as Institutional Review Boards of the Institute of Medical Sciences, Banaras Hindu University, Varanasi, India, and the University of Iowa. All subjects provided written informed consent; in case of illiterate subjects, a thumb print plus a signature of an independent witness were used. For minors under the age of 18, written informed consent was obtained from a parent or guardian. Informed consent procedures for each of the studies were approved by the respective review boards.

## Results

### Study population

Among the total eligible study population of 34,354 subjects, 1,825 were excluded from further analysis because of a reported history of VL prior to the first serosurvey. Thus 32,529 subjects were included in the analysis, of whom 28,073 (86.3%) were also enrolled in a follow-up survey. Participants were divided over study populations and countries as indicated in table 1.

**Table 1:** Baseline sero-status and seroconversion rates in the 4 study cohorts.

Cohort	Number of subjects	Sero-positive at Baseline		Sero-conversion follow-up*	
		DAT	rK39	DAT	rK39
1: KALANET India	7,345	942 (12.8%)	851 (11.6%)	347 (6.2%)	NA
2: KALANET Nepal	5,063	225 (4.4%)	323 (6.4%)	124 (2.9%)	NA
3: TMRC Old area	12,664	800 (6.3%)	678(5.4%)	261 (2.5%)	136 (1.3%)
4: TMRC New area	7,457	735 (9.9%)	556 (7.5%)	254 (4.6%)	453 (7.9%)
All	32,529	2,702(8.3%)	2,408(7.4%)	986 (3.8%)	588 (3.7%)

percentages expressed as a proportion of initial sero-negatives enrolled in second round survey.

Based on the criteria described in the ‘study procedures’ section, cut off values for rK39 ELISA expressed in percentage point optical density of a positive control (pp) varied between cohorts. The cut offs that distinguished seronegative from moderately seropositive individuals in the KALANET cohorts were 27 pp for Nepal and 34 pp for India. The cutoffs for strongly seropositive individuals were 44 and 42 pp respectively. The cut offs for individuals in the TMRC cohorts were lower, i.e., 15 and 21 pp for the ‘old area’, and 11 and 27 pp for the ‘new area’.

### Risk of progression to VL and sero-status at baseline

**Figure 1:**
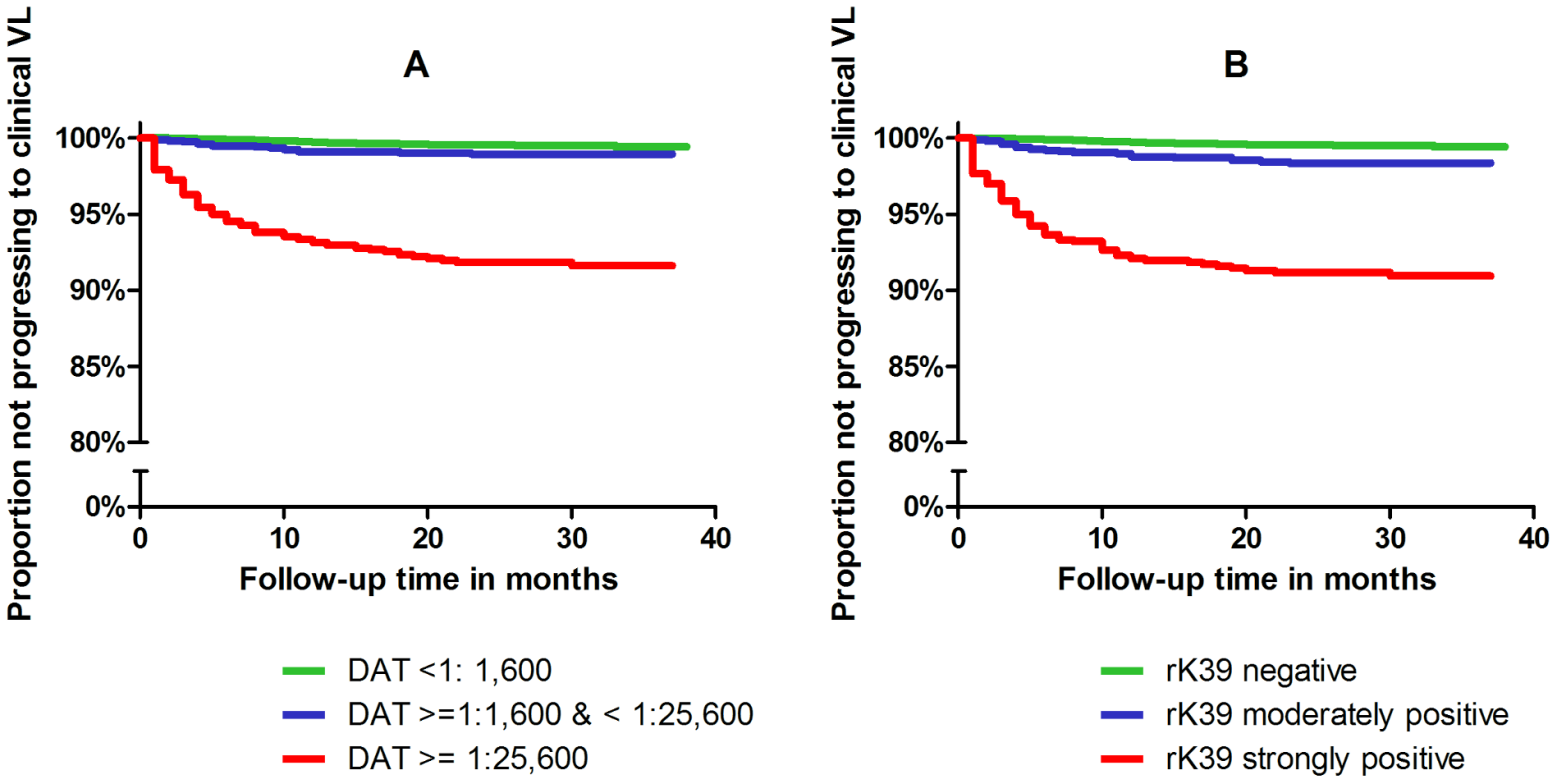
Risk of progression to clinical VL in groups with different levels of (A) DAT and (B) rK39 serologic titers in the baseline surveys. Data from all four cohorts were combined in generating these plots.

Overall 235 VL cases occurred among the72,169 total person years observed, i.e. 3.3 per 1,000 person year at risk. As shown in the Kaplan-Meier graphs in Figure 1, in all cohorts combined the probability of developing VL was strongly increased in individuals with the highest titers of either DAT or rK39.

Because the interactions between titer levels and study cohorts were statistically significant (p = 0.0001 both for DAT and for rK39), hazard ratios are presented for each of the populations separately (Table 2). There was little difference in risk of progressing to clinical VL between individuals who were seronegative for DAT versus individuals in the moderately seropositive group. Only in the cohort 4 (new TMRC area) there was a statistically significant hazard ratio of progression among individuals in the moderately seropositive group (hazard ratio 3.8, 95% CI 1.9–7.3). Among the other cohorts there were either no cases in the moderate titers group or the hazard ratio was very close to 1 when comparing with the seronegative individuals. Among the strongly seropositive individuals however, all hazard ratios were very high and statistically significant, ranging from 7.9 (95% CI 2.3–26.6) in cohort 3 (old TMRC) to 35.6 (95% CI 17.0–74.7) in cohort 2 (KALANET-Nepal) (Table 2).

**Table 2:** Risk of progression to clinical VL in groups of individuals classified as seronegative, moderately seropositive and strongly seropositive according to their baseline DAT titers.

Cohort	DAT titre at baseline	Total evaluated	Number (%) of progressors	Hazard ratio (95% CI)
1:KALANET India	<1∶1,600	6,403	52 (0.8)	ref.
	≥1∶1,600 & <1∶25,600	488	4 (0.8)	1.0 (0.4–2.8)
	≥1∶25,600	454	29 (6.4)	8.2 (5.2–12.8)
2: KALANET Nepal	<1∶1,600	4,838	14 (0.3)	ref.
	≥1∶1,600 & <1∶25,600	82	0 (0)	NA
	≥1∶25,600	143	14 (9.8)	35.6(17.0–74.7)
3: TMRC Old area	<1∶1,600	11,864	20(0.2)	ref.
	≥1∶1,600 & <1∶25,600	571	1(0.2)	1.1 (0.1–7.9)
	≥1∶25,600	229	3(1.3)	7.9 (2.3–26.6)
4: TMRC New area	<1∶1,600	6,722	45(0.7)	ref.
	≥1∶1,600 & <1∶25,600	458	11(2.4)	3.8 (1.9–7.3)
	≥1∶25,600	277	42(15.2)	26.6 (17.4–40.6)

The pattern of progression to VL according to the baseline rK39 status was similar to that according to DAT (Figure 1B and Table 3). Individuals in cohorts 3 and 4 (the two TMRC areas) who were moderately seropositive had a significantly increased risk of progressing to symptomatic disease, although the association was much more pronounced among the strongly seropositive persons. Hazard ratios were very high and highly significant among strongly seropositive individuals in all four cohorts, ranging from 7.7 (95% CI 2.3–26.0) in cohort 3 (old TMRC) to 39.6 (95% CI 25.2–62.3) in cohort 4 (new TMRC) (Table 3).

**Table 3:** Risk of progression to clinical VL by baseline rK39 status.

Cohort	Baseline rK39 status	Total evaluated	Number (%) of progressors	Hazard ratio (95% CI)
1:KALANET India	Negative	6,462	56 (0.9)	ref.
	Moderately positive	565	8 (1.4)	1.6 (0.8–3.4)
	Strongly positive	286	21 (7.3)	9.0 (5.5–14.9)
2: KALANEL Nepal	Negative	4,673	14 (0.3)	ref.
	Moderately positive	155	0 (0)	NA
	Strongly positive	168	13 (7.7)	26.9 (12.6–57.1)
3: TMRC Old area	Negative	11,981	18 (0.2)	ref.
	Moderately positive	410	3 (0.7)	4.9 (1.5–16.8)
	Strongly positive	226	3 (1.1)	7.7 (2.3–26.0)
4: TMRC New area	Negative	6,901	44 (0.6)	ref.
	Moderately positive	376	12 (3.2)	3.6 (1.7–7.6)
	Strongly positive	180	42 (23.3)	39.6 (25.2–62.3)

### Risk of progression to VL and sero-conversion

**Figure 2:**
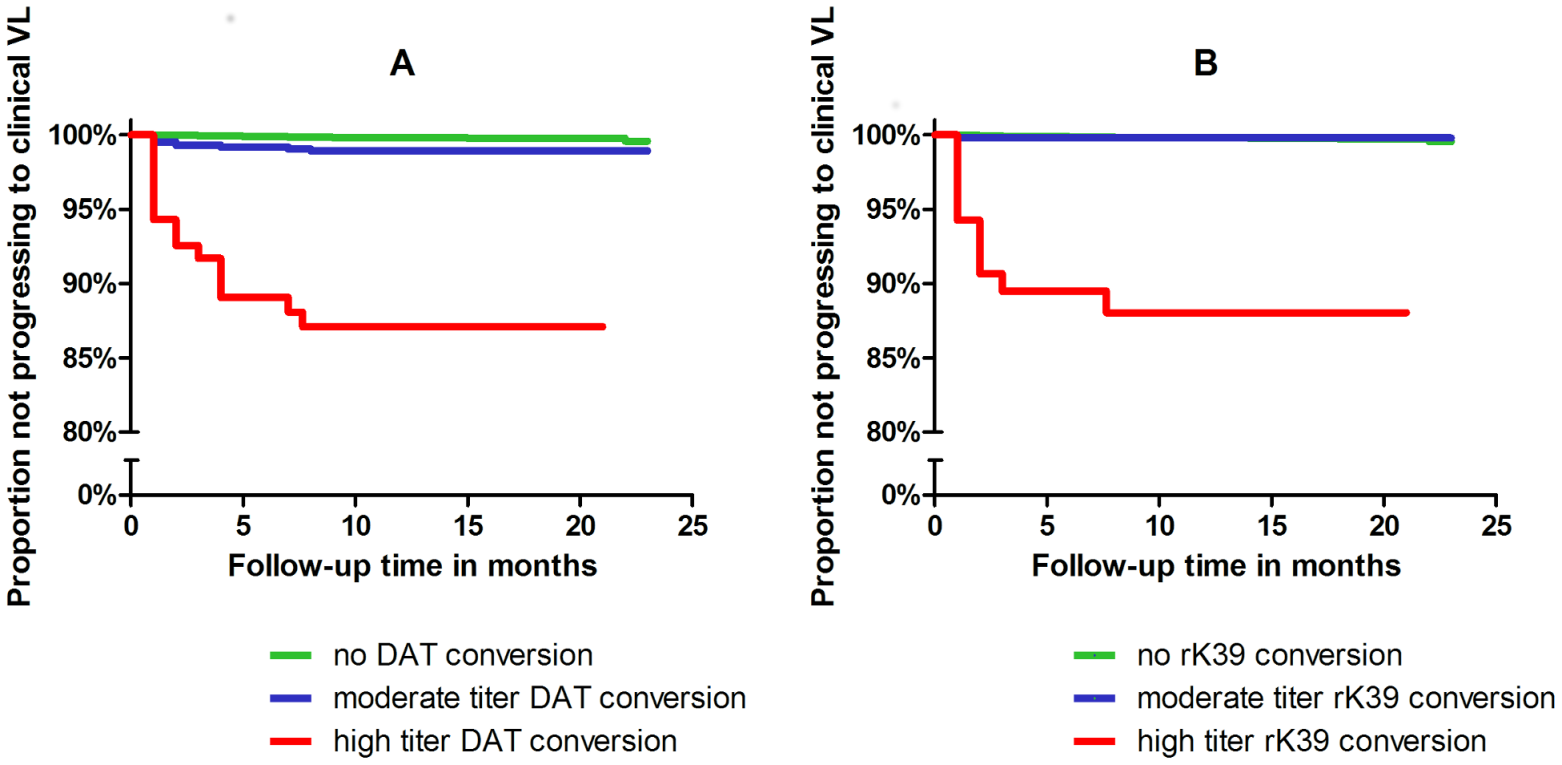
Risk of progression to clinically symptomatic VL according to the class of (A) DAT or (B) rK39 titer at time of seroconversion.

The associations between seroconversion and progression to symptomatic VL were very strong for those with high titers, measured with either DAT or rK39 (Figure 2). The risk of progression to VL among seroconvertors with intermediate titers was not very different from the risk in those who had remained seronegative. As illustrated by the Kaplan-Meier curves in Figure 2, most cases occurred during the first 6 months of follow-up after the second serosurvey.

The overall hazard ratio of developing symptomatic VL when comparing non-convertors to convertors with moderate DAT titers was 6.9 (95% CI 3.4–14.3). Among seroconvertors with high titers the hazard ratio was 97.4 (95% CI 53.5–177.4), with 12% (15 out of 123) of subjects progressing to disease (table 4). There was no significant interaction between cohort and DAT titer level (p = 0.58).

**Table 4:** Risk of progression to symptomatic VL among individuals who seroconverted according to their DAT titer, compared to individuals who did not seroconvert.

Study Population Cohort	DAT Titre at time of 2^nd^ sero survey	Total evaluated	Number (%) of progressors	Hazard ratio (95% CI)
1:KALANET India	<1∶1,600	5,273	8 (0.2)	ref.
	≥1∶1,600 & <1∶25,600	301	3 (1.0)	6.6 (1.8–24.9)
	≥1∶25,600	46	7 (15.2)	111.0 (40.2–306.2)
2: KALANET Nepal	<1∶1,600	4,114	4 (0.1)	ref.
	≥1∶1,600 & <1∶25,600	102	1 (1.0)	10.1 (1.1–90.4)
	≥1∶25,600	22	2 (9.1)	99.2 (18.2–541.5)
3: TMRC Old area	<1∶1,600	10,031	14 (0.1)	ref.
	≥1∶1,600 & <1∶25,600	243	0 (0)	NA
	≥1∶25,600	18	1 (5.6)	44.5 (5.8–340.3)
4: TMRC New area	<1∶1,600	5,326	14 (0.3)	ref.
	≥1∶1,600 & <1∶25,600	217	5 (2.3)	9.0 (3.3–25.1)
	≥1∶25,600	37	5 (13.5)	65.8 (23.6–183.2)
All	<1∶1,600	24,744	40 (0.2)	ref.
	≥1∶1,600 & <1∶25,600	863	9 (1.0)	6.9 (3.4–14.3)
	≥1∶25,600	123	15 (12.2)	97.4 (53.5–177.4)

Hazard ratios are indicated separately for individuals in different classes of DAT titer at the time of seroconversion.

Unlike the data for DAT titers, there was no significant association between moderate titer rK39 conversion and progression to symptomatic VL in the two TMRC cohorts. However there was a very strong and highly significant association with progression to symptomatic VL among those who converted with high rK39 titers. Hazard ratios ranged from 15.9 (95% CI 2.1–121.4) to 123.9 (95% CI 52.7–291.2) among the cohorts (table 5). There was significant effect modification by cohort (p = 0.03), precluding us from calculating an overall effect.

**Table 5:** Risk of progression to symptomatic VL in individuals who seroconverted compared to individuals who did not seroconvert, calculated by groups with low, moderate or high rK39 titers at the time of seroconversion.

Cohort	rK 39 titre at time of 2^nd^ sero survey	Total evaluated	Number of progressors (%)	Hazard ratio (95% CI)
3: TMRC Old area	Negative	10,264	14 (0.1)	ref.
	Moderately positive	85	0 (0)	NA
	Strongly positive	50	1 (2.0)	15.9 (2.1–121.4)
4: TMRC New area	Negative	5,284	13 (0.3)	ref.
	Moderately positive	416	1 (0.2)	0.9 (0.1–7.1)
	Strongly positive	37	9 (24.3)	123.9 (52.7–291.2)

Please note: Individuals in the KALANET study (cohorts 1 and 2) are not included in this analysis of seroconversion because their serologic status was measured only once.

## Discussion

This study analyzed data from four large cohorts of individuals living in regions endemic for *L. donovani* infection in India and Nepal. The data revealed strong associations between the magnitude of positive serology, measured by DAT and/or rK39, and the risk of progressing to symptomatic VL. A documented recent seroconversion was also associated with a risk of developing symptomatic VL. These associations were consistent and statistically significant among individuals with high titers of either serological marker. Among those seroconverting to high titers the risk of progressing to disease was particularly strong, reaching a hazard ratio of 97.4 among individuals with high DAT titers. The relatively long intervals between surveys (6–12 months) must have caused us to miss some seroconversions among later VL cases, which would lead to an underestimation of the true hazard ratio. Even though the strengths of the associations varied between cohorts and between markers, consistent patterns were observed across the four cohorts and the two serologic markers.

With the exception of individuals who seroconverted according to DAT titer, variations between the strengths of associations precluded us from being able to calculate overall effects including all four cohorts. These variations were probably due to chance, since the numbers of subjects in each of the titer level groups considering all seropositive persons or all individuals who seroconverted was not very high. As a result, the absolute numbers of individuals developing VL were low especially in the groups with moderately positive titers. Overall fewer than 1 in 10 infected persons developed symptomatic disease. Another possible source of variation could be the positive predictive value of the tests for detecting infection, which varies with prevalence. However the variation observed could also be explained by the epidemiological status of individuals in each of the cohorts. VL tends to cause micro-epidemics that saturate an area and then “move on” to a neighboring area [24]. If an area is at the end of the epidemic wave many inhabitants will be seropositive but few additional cases will arise; if the outbreak is still at the beginning there will be fewer seropositive individuals and a larger proportion of seroconverting persons will progress to disease. Thus, cohorts belonging to the KALANET and old area TMRC studies were selected based on a reported high incidence of VL over the 3 preceding years. However the new area TMRC cohort was selected based on a high incidence of VL during the 12 months preceding the survey. This difference may explain the higher proportion of subjects progressing to disease in the TMRC new area.

Contrary to what has been reported in some smaller cohort studies and among studies of other *Leishmania* species, our study populations exhibited a strong association between serological markers and progression to symptomatic VL among asymptomatic individuals [16] [17] [18]. Among the 235 VL cases that developed in our 32,529-person strong study population, 88 arose from a subgroup of 1,103 individuals with high initial DAT titers. Even using a very low cut-off for a positive DAT titer (1∶800), Sundar *et al.* found no seropositive individuals among 100 Indian controls living in non-endemic regions, providing further evidence for the fact that positive serology is a result of infection with VL parasites and not due to cross reactivity [25]. Although the vast majority of individuals who are seropositive do not progress to disease, seroprevalence and seroconversion remain good markers for prevalence and incidence of *Leishmania* infection in epidemiological studies, and as tools to monitor the impact of interventions, especially when high titer cut offs are used.

The potential public health impact of asymptomatic VL infection remains undefined. There are substantial numbers of asymptomatic seropositive individuals in areas of Bihar and Nepal with endemic VL. According to the current study these individuals are at increased risk of progression to disease. The fact that 15.2% of individuals in the new TMRC area with high positive DAT titers progressed to symptomatic VL raises the question of whether prophylactic treatment should be considered. Prophylactic treatment for tuberculosis is recommended for persons with a positive tuberculin skin test, particularly if skin test conversion is recent. The lifetime risk of progression from tuberculosis infection to disease among non-immunodeficient hosts however is no more than 10%, half of which occurs in the first years following infection [26] [27]. Similar to treatment of individuals with a positive tuberculin skin test, “prophylactic” treatment for VL would constitute treatment of low-level asymptomatic infection to lower the microbial burden. Persons with a latent *M. tuberculosis* infection are not important as sources of transmission, whereas it is still a subject of debate whether individuals with asymptomatic infection constitute a reservoir impacting the incidence of VL [13]. Thus the current study underscores a need for two follow-up investigations. First, there is a need for xenodiagnosis studies to explore whether subclinically infected individuals, preferably stratified by antibody titer levels, can transmit infection to sand flies [28]. Second, research is needed to explore the clinical benefit and the potential public health impact of treating individuals with asymptomatic infections with the parasites causing VL. In combination such a series of studies could lead to significant policy changes in the control of visceral leishmaniasis and eventually enhance its impact.

### Conclusion

At population level, specific antibodies against *L.donovani* as measured in DAT or rK39 are strongly associated with risk to disease progression. This relationship is even more pronounced among individuals who have recently seroconverted.

## Supporting Information

Checklist S1 STROBE checklist.
(DOC)
