# Supplementary material for: Strong Association between Serological Status and Probability of Progression to Clinical Visceral Leishmaniasis in Prospective Cohort Studies in India and Nepal
Source: PLoS Negl Trop Dis. 2014 Jan 23;8(1):e2657. doi: 10.1371/journal.pntd.0002657 (PMC3900391; doi:10.1371/journal.pntd.0002657)
Supplement: Checklist S1 — STROBE checklist. (DOC) [file pntd.0002657.s001.doc]

STROBE Statement—checklist of items that should be included in reports of observational studies

|  | | Item No | Recommendation |
| --- | --- | --- | --- |
| **Title and abstract** | | 1 | √ (*a*) Title mentions ‘prospective cohort studies’ |
|  |  |  | √ (*b*) see abstract |
| Introduction | | | |
| Background/rationale | | 2 | √See introduction |
| Objectives | | 3 | √See introduction, last paragraph |
| Methods | | | |
| Study design | | 4 | √See ‘study procedures’ section |
| Setting | | 5 | √See ‘study populations’ section |
| Participants | | 6 | √ (a)See ‘study populations’ section |
|  |  |  | (b)Not applicable |
| Variables | | 7 | √See ‘study procedures’ section |
| Data sources/ measurement | | 8* | √ See ‘study populations’ section |
| Bias | | 9 | We combine data from four population based cohorts and identify trends that are consistent between these cohorts. |
| Study size | | 10 | We used available data on existing cohorts |
| Quantitative variables | | 11 | √ See ‘study procedures’ section |
| Statistical methods | | 12 | √ (*a*) See ‘data analysis’ section |
|  |  |  | √ (*b*) See ‘data analysis’ section |
|  |  |  | (*c*) Not applicable |
|  |  |  | (*d*) See ‘data analysis’ section |
|  |  |  | √ (*e*) We compared findings between 4 different cohorts, see ‘study populations’ section |
| Results | | | |
| Participants | 13* | √ (a) See ‘Results’ section, first paragraph | |
|  |  | (b) Not relevant | |
|  |  | (c) Not relevant | |
| Descriptive data | 14* | √ (a) See ‘Study population’ in ‘Results’ section | |
|  |  | √ (b) See ‘Study population’ in ‘Results’ section | |
|  |  | √ (c) See ‘Risk of progression to VL and sero-status at baseline’ in ‘Results’ section | |
| Outcome data | 15* | √ See ‘Results’ section, proportions of progressors to clinical VL and corresponding hazard ratios are listed in table 2-5. | |
| Main results | 16 | (*a*) Not applicable | |
|  |  | √ (*b*) Category boundaries mentioned in ‘Results’ section are explained in ‘study procedures’ section | |
|  |  | (c) We provide both the absolute risk and the hazard ratios. | |
| Other analyses | 17 | √See ‘Results’ section | |
| Discussion | | | |
| Key results | 18 | √See paragraph 1 of ‘Discussion’ section | |
| Limitations | 19 | √ See ‘Discussion’ section, 2^nd^ half of first paragraph | |
| Interpretation | 20 | √ See ‘Conclusion’ section | |
| Generalisability | 21 | √ See paragraph 3 of ‘Discussion’ section | |
| Other information | | | |
| Funding | 22 | √ The KALANET community trial was part of a large project conducted by the KALANET consortium funded by the European Union under its 6th 319 Framework Programme (INCODEV/320 Project 015374).  The TMRC study is supported in part with funds from Tropical Medicine Research Center P50 AI-074321 (all authors) from the US National Institutes of Health, grants R01 AI076233 and  NIAID R01 AI045540  from the NIH (MEW) and a VA Merit Review grant (MEW).  The content is solely the responsibility of the authors and does not necessarily represent the official views of the National Institutes of Health. | |

*Give information separately for cases and controls in case-control studies and, if applicable, for exposed and unexposed groups in cohort and cross-sectional studies.

**Note:** An Explanation and Elaboration article discusses each checklist item and gives methodological background and published examples of transparent reporting. The STROBE checklist is best used in conjunction with this article (freely available on the Web sites of PLoS Medicine at http://www.plosmedicine.org/, Annals of Internal Medicine at http://www.annals.org/, and Epidemiology at http://www.epidem.com/). Information on the STROBE Initiative is available at www.strobe-statement.org.
